# INCREASED CARDIOVASCULAR DISEASE RISK FACTORS IN PERSONS WITH CEREBRAL PALSY

**DOI:** 10.1093/geroni/igz038.1277

**Published:** 2019-11-08

**Authors:** Patricia C Heyn

**Affiliations:** University of Colorado Denver Anschutz Medical Campus, Aurora, Colorado, United States

## Abstract

Cardiovascular disease (CVD) remains the leading cause of global mortality. Individuals with disabilities are at increased risk of CVD in part due to their musculoskeletal and/or cognitive impairments that can challenge their participation in physical activities. Gait ability has been associated to several key health outcomes including morbidity. Unfortunately, there is little research done to understand how individuals with obstructed mobility (i.e. developmental or injury condition) grow older. Our research team (Heyn et al 2019), evaluated the prevalence of CVD risk factors and age-related health outcomes associated to aging on a cohort of adults with cerebral palsy with obstructed mobility. Metabolic syndrome was identified in 17.1% of the cohort, higher than the 10% in the NHANES sample. CVD risk factors were much higher in this cohort as compared to normative population data. There was a positive correlation between mobility level, waist circumference (r=0.28, p=0.02), and waist-to-hip ratio (r=0.28, p=0.02).

